# Predictors and Moderators of Outcomes in a Trial of Cognitive Behavioural Therapy Integrated with Behavioural Weight Loss for High Weight Individuals with Disorders of Recurrent Binge Eating

**DOI:** 10.3390/nu17071288

**Published:** 2025-04-07

**Authors:** Haider Mannan, Marly Amorim Palavras, Angelica Claudino, Phillipa Jane Hay

**Affiliations:** 1 Translational Health Research Institute, School of Medicine, Western Sydney University, Campbelltown, NSW 2560, Australia; h.mannan@westernsydney.edu.au (H.M.); marlypalavras@gmail.com (M.A.P.); 2 Eating Disorders Program (PROATA), Department of Psychiatry, Universidade Federal de São Paulo (UNIFESP), Rua Major Maragliano 241, São Paulo 04017, SP, Brazil; 3 Mental Health Services, South Western Sydney Local Health District, Liverpool, NSW 2170, Australia

**Keywords:** loss of control, overeating, mid-therapy, eating disorders, predictors, moderators

## Abstract

**Background/Objectives**: To inform person-centred clinical practice, it is important to know what features may predict or moderate treatment outcomes. Thus, we investigated pre-treatment clinical features and mid-therapy reduction in loss of control over eating (MTLOCE), including impacts on treatment outcomes of a new manualised psychotherapy, a healthy approach to weight management and food in eating disorders (HAPIFED). HAPIFED was developed as an integrated psychological and behavioural treatment for individuals with bulimia nervosa or binge eating disorder, which are co-morbid with a high body mass index (BMI). **Methods**: In total, 50 participants were randomised to HAPIFED and 48 were randomised to the control cognitive behaviour therapy-enhanced group. Assessments included mental health-related quality of life (MHRQoL), eating disorder symptom severity, binge-eating frequency, BMI, and loss of control over eating (LOCE) at baseline, mid-treatment, end-treatment, and 6 and 12 months end of follow-up (EndFU). These were measured with the SF-12, the EDE-Q, and the LOCES, respectively. Linear and negative binomial mixed models were used. Missing data were imputed multiple times, assuming intention of treatment for the analysis. **Results**: Pre-treatment eating disorder symptom severity, MHRQoL, and BMI positively predicted eating disorder symptom severity, MHRQoL, and BMI up to 6 and 12 months end of follow-up. Mid-treatment LOCE MTLOCE predicted improved MHRQoL (coefficient = 0.387, 95% CI 0.0824–0.6921, *p* = 0.004), reduced binge-eating frequency (IRR = 0.5637, 95% CI 0.3539–0.8977, *p* = 0.0191), and eating disorder symptom severity (coefficient= −0.65, 95% CI −1.0792–−0.2217, *p* = 0.0139). Neither purging nor illness duration were a significant predictor of any of the outcomes. The effect of HAPIFED was not moderated by baseline weight/BMI but was moderated negatively by MTLOCE for binge-eating frequency (coefficient = −0.636, SE = 0.28, *p* < 0.05, IRR = 0.529) and eating disorder symptom severity (coefficient = −0.268, SE = 0.13, *p* < 0.05, Cohen’s d = −0.102). **Conclusions**: Greater control over eating improved MHRQoL and decreased the frequency of binge-eating episodes and eating disorder symptom severity. These positive effects were moderated by being in the HAPIFED group, supporting previous findings of benefits to people’s mental health through participation in the HAPIFED trial.

## 1. Introduction

The management of high weight in people with eating disorders lacks evidence. Whilst leading treatments such as cognitive behavioural therapy (CBT) [1] are effective for the treatment of eating disorders in adults [2,3,4], they are not weight loss treatments. Furthermore, problematic recurrent binge eating, the defining feature of eating disorders such as bulimia nervosa (BN) and binge-eating disorder (BED) [5], is common (ranging from 13 to 78%) in people with higher weight seeking treatment [6]. Evidence also shows that the greater the weight, the greater the severity of binge-eating [7], and in the general community, binge eating is increasing at a higher rate where it is experienced alongside an increase in body weight [8].

In an attempt to bridge the gap in evidence for treatment for individuals with both recurrent binge-eating symptoms and high weight, a multidisciplinary manualised psychological therapy [9] was developed. The purpose of the healthy approach to weight management and food in eating disorders (HAPIFED) was to integrate the central features of CBT-enhanced (CBT-E), evidence-based psychological therapy for BN and BED [1], and the psychobehavioural strategies for weight loss found in behavioural weight loss therapy (BWLT), including lifestyle changes [10]. This was an innovative intervention tested and compared to original CBT-E (control group) in a single-blind randomised controlled trial (RCT) [9]. The results showed no differences in between-group comparisons for the weight loss measures and for eating disorder symptoms overall [9]. However, HAPIFED was favoured for secondary outcomes of the absence of purging behaviour reported at the eating disorder examination interview [11] (*p* = 0.016) and binge-eating remission rates specifically at 12-month follow-up (*p* < 0.05) [9]. As in other studies (e.g., [12]), overall recovery rates or remission were under 40%.

It is important to better understand which features may be associated with a poorer or better outcome in treatments for people experiencing the high physical and mental health burdens of BN or BED and high weight [13,14,15]. These include pre-treatment variables that may interfere with the treatment response despite the treatment type received (predictor variables) and the variables that may intersect with the intervention and influence the outcomes (moderator variables) [16]. In BN and BED treatments, at baseline, the frequency of binge/purge behaviours has been reported to predict worse results at the end of treatment and follow-up [17]. However, in this systematic review, lower levels of eating disorder symptomatology predicted a better outcome in a sample with BN [17]. An early reduction in binge eating is one of the few moderating or mediating features found to be consistently associated with a better outcome [17,18,19].

In the past decade, there has been increased recognition of the role of LOCE as a key feature of binge eating, and it is the defining feature of binge eating in the ICD-11 criteria [20]. LOCE has also been found to be a transdiagnostic feature across a range of obesity-related disordered eating behaviours, including BED and BN [21]. LOCE may be expressed in diverse ways, of which the most well-known are the two descriptors in the DSM-5, namely “It’s hard for me to stop eating when I eat like that” and “I feel like I can’t stop or limit the amount of food or the type of food I’m eating”. It may also be experienced as “abandoned efforts” to control inevitable eating [5]. To our knowledge, the examination of prediction and moderation by LOCE on the treatment outcomes has not been conducted, and it is not known whether a change in LOCE may moderate treatment outcomes independent of binge-eating episodes.

Thus, the present study aimed to investigate features that may predict and moderate either the weight loss or eating disorder symptom outcomes in the HAPIFED controlled trial. The exploratory hypotheses were that (a) independent variables, e.g.,illness duration, binge eating frequency, eating disorders symptom severity, purging behaviour, MHRQoL, LOC over eating, weight, and BMI, would all be measured at baseline and also a mid-therapy reduction in loss of control over eating (MTLOCE) may predict the 12-month follow-up outcomes, i.e., binge-eating frequency, eating disorders symptom severity, BMI, and MHRQoL, and (b) all the independent variables presented above may moderate the efficacy of HAPIFED.

## 2. Materials and Methods

### 2.1. Design

A single-blind, two-arm RCT was conducted as part of the Eating Disorders Program (PROATA) of the Department of Psychiatry of the Universidade Federal de São Paulo (UNIFESP), Brazil (clinicaltrials.gov registration number NCT02464345), and ethical approval was provided by the Human Research Ethics Committee of UNIFESP/Brazil, number CAAE 43874315.4.0000.5505 on 14 May 2015.

### 2.2. Participants and Procedures

The recruitment took place from July 2015 to November 2017 through the PROATA’s waiting list, printed media, and advertisements on the radio. Ninety-eight participants fulfilled the inclusion criteria, which were age ≥ 18 years old, either sex, threshold or subthreshold criteria for BN or BED according to DSM-5 [5] and/or ICD-11 criteria [20], and a BMI ≥ 27 and <40 kg/m^2^. Both the DSM and ICD require recurrent binge eating at minimum frequency and duration, i.e., weekly for 3 months in DSM and one month in ICD, for a diagnosis of BN or BED. Additional criteria for BN are the presence of regular behaviours that compensate for binge eating, which include purging, fasting, and/or driven exercise, and body image concerns of weight/shape overvaluation. The ICD criteria also allow for subjective (not objectively large) binge-eating episodes. For BED, both DSM and ICD require binge eating to be associated with marked distress, and the DSM has additional 3/5 mandatory specifiers of binge eating, such as eating more rapidly, when not hungry, until uncomfortably full, alone because of embarrassment, and experiencing negative emotions such as disgust or guilt (Criterion B). The exclusion criteria were currently receiving psychological treatment for eating disorders, use of weight loss medication, current diagnosis of psychosis or bipolar disorder and/or a high level of suicide risk, history of bariatric surgery, and clinical conditions that disturb appetite regulation (e.g., Prader–Willi, Cushing’s syndrome, etc.). The eligible participants attended an in-person interview to receive the details about the trial, to sign the informed consent form, to answer a psychiatric anamnesis, and to complete self-report questionnaires. Those eligible were invited to a second interview to confirm eating disorder diagnoses and symptomatology (for more details, see [9]). After diagnostic confirmation, they were allocated through a computer-generated and externally administered randomisation process. Only the main team of researchers and the therapists had access to this allocation. The participants remained blind to their allocation until the end of the trial and follow-up, i.e., 18 months after baseline.

In total, 66 (67.3%) and 13 (13.3%) participants met the DSM-5 diagnostic criteria for BED and BN, respectively. The remaining 19 participants were a mixture of other specified feeding or eating disorders: (OSFED) BED-type (*n* = 5, 5.1%), OSFED BN-type (*n* = 7, 7.1%), and unspecified feeding or eating disorder (UFED) (*n* = 7, 7.1%). Of the latter group, although all reported regular recurrent binge eating, some did not meet the criteria for BED or BN. Five participants met neither criterion B nor criterion C (marked distress) for BED, one met all but criterion D for BN, and one met all criteria for BN, but their binge-eating episodes were subjective only.

### 2.3. Interventions

The manualised experimental intervention HAPIFED [9] was delivered in 30 sessions, the first an individual session followed by 29 in a group format over 6 months. During the first four weeks, the sessions were delivered twice a week and, after that, once a week. HAPIFED integrates behavioural weight loss with CBT-E and has five stages offering psychoeducation, self-monitoring, behavioural change, cognitive reframing of unhealthy beliefs and attitudes related to eating disorders behaviours, and the development of a long-term plan to deal with the relapses. The manualised control intervention, CBT-E [1], is considered the intervention with probably the best evidence for BN and BED [2,3,4]. A broad version of CBT-E was delivered to match HAPIFED for a number of sessions and durations. CBT-E is organised in four stages, also offering psychoeducation, self-monitoring, and a review of maintaining mechanisms (e.g., mood intolerance), with the same termination sessions as HAPIFED.

HAPIFED differs from CBT-E in seven areas: (1) psychoeducation topics include eating disorders and weight management, (2) nutritional support is over 4 sessions and is provided by a dietitian, (3) hunger and satiety ratings are monitored, (4) a multidisciplinary team is employed, (5) weight management is provided, (6) behavioural activation is provided, and (7) physical activities are guided by an occupational therapist who visits the house’s participant twice during the intervention. These are presented in Supplementary Table S1.

After six months of the intervention, all participants were followed up for one year. In the first six months of follow-up, they received 4 sessions led by the same intervention therapists, and in the final six months, one more session (for more details, see [9]).

### 2.4. Assessment

The assessment occurred at five time points: baseline, middle of treatment (3 months), end of treatment (6 months), 6-month follow-up, and 12-month follow-up, and included those who did not complete the treatment. For the purpose of this study, the assessment comprised the following measures:

1. Socio-demographic data (age, sex, race, marital status, occupation, and education level) were collected through a self-reported questionnaire at baseline.
2. An objective measure of weight and height was obtained with a calibrated scale and a stadiometer for the calculation of BMI (kg/m^2^) in the five time points mentioned above.
3. The widely used and well-validated eating disorder examination (EDE) interview (17th Edition [11]) was used to assess eating disorder diagnoses and symptomatology, global eating disorder symptoms severity, and binge-eating frequency at baseline, end of treatment, and 6 and 12-month follow-ups. It has a global score of general eating disorder symptoms, which is derived from four subscale scores of restraint, eating concern, weight concern, and shape concern. The other relevant variables for this study are binge-eating frequency and compensatory (e.g., purging) methods. Cronbach’s α for the global score was 0.77 (22 items).
4. The 28-item eating disorder examination questionnaire (EDE-Q; version 6) is derived from the EDE and was used to assess the eating disorder symptom severity over a one-month period at all time points [22,23,24]. It is a reliable and valid measure [24] and has a global score of general eating disorder symptoms, which is derived from four subscale scores: restraint, eating concern, weight concern, and shape concern. In the present study, Cronbach’s α for the global score was 0.81 (29 items).
5. The loss of control overeating scale (LOCES) 24-item Portuguese translation was used to assess the self-reported presence of LOC over eating in the last four weeks in all five time points [25,26]. It uses a 5-point scale from never to always experiencing listed features of LOCE. LOCES assesses the diverse expressions of LOCE and has robust psychometric properties]. In particular, the two descriptors of LOCES based on DSM-5 are “It’s hard for me to stop eating when I eat like that” and “I feel like I can’t stop or limit the amount of food or the type of food I’m eating”. Cronbach’s α for the current sample was 0.92 (24 items).
6. The 12-item short form health survey (SF-12 version 1) evaluated the health-related quality of life in its physical and mental health features in all five time points [27,28]. For the purpose of this paper, only MHRQoL data were considered. It has been validated in populations of people with mental ill-health [29]. Cronbach’s α was 0.78 for the seven mental component summary items.

### 2.5. Outcomes

The outcomes were frequency of binge-eating episodes, eating disorder symptom severity (measured by the EDE-Q global score), BMI, and MHRQoL (measured by the SF-12), all at the provided time points.

### 2.6. Statistical Analysis

Generalised linear mixed models were used to determine the predictors and moderators of the treatment outcomes over all time points up to the 12-month follow-up. For the frequency of binge-eating episodes as the outcome, the link function was logged, and the error distribution was assumed to follow a negative binomial (thus, if the IRR < 1, the relationship was negative). For eating disorder symptom severity, BMI and MHRQoL outcomes were identity and normal distribution, respectively. The predictors examined were baseline measures of frequency of binge-eating episodes, eating disorder symptom severity, illness duration, purging behaviour, MHRQoL, weight and BMI (only when the outcome was BMI at follow-up), and MTLOCE. It is noted that weight was a predictor in all models except for the BMI model. There was a baseline imbalance between the treatment and control groups by age, which prompted us to control for this variable in our regression models. HAPIFED and the type of eating disorder diagnosis were considered the other control variables. The latter was considered in order to reduce the contribution of heterogeneity in the case of a mix of eating disorder types in the study sample based on the regression estimates. The effect sizes used for assessing the strength of predictors (continuous) were the partial regression coefficient and incidence rate ratio when the outcome was continuous and counted, respectively. The effect sizes used for assessing the strength of moderators (continuous) with HAPIFED were Cohen’s d and the incidence rate ratio (IRR) when the outcome was continuous and counted, respectively. These effect sizes were only estimated when the respective predictor was found to be significant. The guidelines suggested by Rosenthall and Rubin [30] were used for classifying the effect sizes into small, moderate, and large. Missing data were estimated using multiple imputations with the MCMC (Markov chain Monte Carlo) algorithm (multivariate normal imputation). Multivariate normal imputation (MVNI) assumes that observed data and missing data follow a joint multivariate normal distribution and that missing data are missing at random (MAR). MVNI can be robust to violations of the multivariate normal assumption, especially when the amount of missing data is not excessive (such as in our analysis). However, we found that this assumption was not violated. We also observed no systematic missingness or missingness not at random. The estimates were pooled using Rubin’s rules [30,31]. All analyses were performed using SAS version 9.4 software [32].

## 3. Results

### 3.1. Descriptive Analysis

Nearly all the 98 participants included in this study were women (*n*= 94, 96%), most were Caucasian (*n* = 73, 74.5%) and employed (*n* = 59, 60.2%), 41% were married (*n* = 44), and 43% (*n* = 42) completed tertiary education. The mean values for baseline age, weight, and BMI were 40.55 years (SE 1.18), 89.26 kg (SE 1.27), and 33.68 kg/m^2^ (SE 0.33), while the baseline weight range was 61.9–114.8 (see Supplementary Figure S1 for the participant flow).

Table 1 shows the mean (SE) values for all predictor variables considered at baseline, including illness duration, binge-eating frequency, eating disorders symptom severity score, MHRQoL, LOC over eating, weight, BMI, and purging behaviour, and the MTLOCE variable for the whole sample (*n* = 98).

### 3.2. Predictors of Treatment Outcomes at 12-Month Follow-Up

There was a decline in LOCE from baseline (mean baseline LOCE score = 3.21. SE = 0.06) to mid-therapy (mean MTLOCE score = 2.32, SE = 0.09). With binge-eating episode frequency as the dependent variable, the significant predictors were MTLOCE (*p* = 0.0163) and baseline eating disorder symptom severity scores (*p* = 0.0293). One unit greater in MTLOCE was associated with a 0.5637 times lower (95% CI 0.3539 to 0.8977) incidence rate ratio of binge-eating episode frequency. This means that binge-eating episode frequency decreased by 43.63% with one unit greater MTLOCE. Thus, if a participant reported a reduction from always experiencing LOCE to often experiencing LOCE and had a frequency of binge-eating episodes of 10 times a week, this frequency would reduce to around 5.64 episodes a week. One unit greater baseline eating disorder symptom severity was associated with a 1.48 times higher (95% CI 1.0413 to 2.1075) incidence rate ratio of binge-eating episode frequency. There was a statistical trend for baseline MHRQoL (*p* = 0.06) in the prediction of the frequency of binge-eating episode frequency.

With eating disorder symptom severity as the outcome, the significant predictors were MTLOCE (*p* = 0.004) and baseline eating disorder symptom severity (*p* = 0.0139). One unit greater MTLOCE was associated with a 0.65 unit decrease (95% CI −1.0792 to −0.2217) in eating disorder symptom severity. One unit greater baseline eating disorder symptom severity score was associated with a 0.387 unit increase (95% CI 0.0824 to 0.6921) in eating disorder symptom severity.

With BMI as the outcome, the only significant predictor was baseline BMI (*p* < 0.001). One unit increase in baseline BMI was associated with a 0.9092 unit increase (95% CI 0.7456 to 1.0728) in BMI.

With MHRQoL as the outcome, MTLOCE (*p* = 0.004) was positively associated, as one unit greater MTLOCE increased the outcome by 5.623 units (95% CI 1.6877 to 9.5587). Another significant (*p* = 0.0127) predictor was baseline MHRQoL. One unit increase in baseline MHRQoL was associated with a 0.3528 unit increase (95% CI 0.0789–0.6266) in MHRQoL.

There was a statistical trend for baseline binge-eating episode frequency to be associated with MHRQoL (*p* = 0.07). Purging and illness duration were a significant predictor for none of the outcomes. All results are shown below in Table 2.

### 3.3. Moderators of Treatment Outcomes at 12-Month Follow-Up

There was moderation in the effect of HAPIFED on binge-eating episode frequency by MTLOCE (slope = −0.636, SE = 0.28, *p* < 0.05, IRR = 0.529), but not by baseline BMI (slope = 0.009, SE = 0.02, *p* > 0.05). This meant that for those assigned to the HAPIFED group, binge-eating episode frequency decreased by 46.4% with every one-unit greater MTLOCE. The effect size was determined as moderate using Rosenthal and Rubin’s [30] criteria.

Moderation was also found in the association of HAPIFED therapy with an eating disorder symptom severity score by MTLOCE (slope = −0.268, SE = 0.13, *p* < 0.05) with a small effect size (Cohen’s d = −0.102) using Rosenthal and Rubin’s [30] criteria. This meant that for those assigned to the HAPIFED group, the frequency of an eating disorder symptom severity score decreased by 73.2% with every one-unit greater MTLOCE. However, there was no moderation by baseline weight (slope = −0.008, SE = 0.02, *p* > 0.05).

MTLOCE did not moderate the association between HAPIFED and MHRQoL (slope = 1.944, SE = 4.05, *p* > 0.05). In addition, baseline weight did not moderate the association between HAPIFED and MHRQoL (slope = 0.208, SE = 0.90, *p* > 0.05). There was no moderation in the effect of HAPIFED therapy on BMI by MTLOCE (slope = 0.456, SE = 0.75, *p* > 0.683) or baseline BMI (slope = 0.032, SE = 0.15, *p* > 0.05). All results are shown in Table 3.

## 4. Discussion

This study investigated predictors and moderators of outcomes longitudinally following HAPIFED, integrated CBT-E, and BWLT for treating BED or BN in a clinical population with a high BMI. MTLOCE was the novel predictor and moderator investigated. The main findings of this study were the following: (a) the baseline lower eating disorder symptom severity scores predicted a reduction in the binge-eating episodes frequency and eating disorder severity scores at 12-month follow-up; (b) a higher MHRQoL at baseline predicted improved MHRQoL at 12-month follow-up; (c) a higher BMI at baseline predicted higher BMI at 12-month follow-up. These findings are consistent with prior research. An interesting and new finding was that greater MTLOCE positively predicted MHRQoL and, hence, was a protective factor for this outcome. It was also found that MTLOCE negatively predicted the frequency of binge-eating episodes and eating disorder symptom severity, being, consequently, a protective factor of these two outcomes. These associations between MTLOCE and certain treatment outcomes remained after adjusting for the baseline frequency of binge-eating episodes. In addition to being a predictor variable, MTLOCE was also found to be a moderating variable, i.e., it moderated the decrease in binge-eating episode frequency and eating disorder symptom severity effects of HAPIFED but did not moderate weight loss. Overall, our findings only partially supported the exploratory hypotheses that baseline eating disorder symptom severity, binge-eating episode frequency, purging, illness duration, BMI, and MHRQoL may predict the frequency of binge-eating episodes, eating disorder symptom severity, BMI, and MHRQoL, but that MTLOCE may predict and moderate treatment outcomes. The following paragraphs discuss these results in the context of prior research.

The present findings concerning predictors are consistent with findings in treatment trials of CBT-E or other treatments for BED or BN. In a systematic review with mixed samples and treatments (e.g., CBT, inpatient or day hospital, BWLT, or pharmacotherapy, etc.), Vall and Wade [17] found a poorer outcome in individuals with more frequent binge/purge episodes at baseline and better outcomes for those with BN and lower levels of eating disorder symptoms at baseline. Masheb and Grilo [33], in a 12-week RCT comparing CBT versus BWLT using a guided self-help approach in a BED sample, found that binge frequency was predicted by baseline binge frequency and eating disorder psychopathology, and eating disorder symptoms were also predicted by baseline eating disorder psychopathology. Baseline measures such as objective binge eating and/or purge behaviour, BMI, and weight loss were predictor variables in eight studies of participants with BN in the Linardon systematic review [18], albeit there was little consistency in findings.

There is little literature regarding the relation between MHRQoL, disorders of recurrent binge eating, and high weight, and the majority of the studies relate to bariatric surgery. In a prospective observational study with Spanish adult women receiving outpatient treatment in a general university hospital, data regarding health-related quality of life were collected from a sample of 124 participants with an eating disorder diagnosis [34]. At the 6-month follow-up, the BED group (*n* = 11) showed the highest score in the mental domain (indicating a better MHRQoL), followed by the BN group (*n* = 35), OSFED group (*n* = 19), and the anorexia nervosa (AN) group (*n* = 59) (*p* = 0.006). In the BED group, age was the only significant predictor of MHRQoL. Lam et al. [35] found that higher baseline MHRQoL predicted a better MHRQoL at 12-month follow-up in a sample of adults with a BMI ≥ 40 kg/m^2^ receiving a multidisciplinary weight management program. Thus, across diverse samples of people with recurrent binge eating and high weight, a better quality of life at baseline was found to favour a better quality of life at the end of treatment.

This paper offers innovative data regarding moderators of treatment, considering the interaction between the novel therapy HAPIFED and MTLOCE and the significant findings of an existing correlation between this interaction and the outcomes of binge-eating episode frequency and eating disorder symptom severity scores. Jenkins et al. [36] found that binge-eating episodes negatively impact quality of life, and the present study suggests that LOCE, more than the size of the amount eaten, during the binge-eating episode is associated with lower quality of life. This supports the positive effects of our findings in the HAPIFED trial [9]. Moreover, the systematic review investigating LOCE as a transdiagnostic characteristic in obesity-related eating behaviours [21] found a positive correlation between LOCE and binge-eating behaviour. These authors also attested that LOCE may compromise the MHRQoL. Findings from Bodell et al. [37] also support a high correlation between LOCE and eating disorder symptomatology and, in particular, binge-eating episode frequency. Finally, in an RCT of liraglutide for the treatment of BED, researchers examined whether the size of the binge (largest) or the degree of LOCE (in an independent correlation) were associated with clinical features of BED in participants [38]. The results of this study showed independent correlations between higher LOCE and binge size with eating disorder symptom severity and lower general quality of life. Taken together with the present study findings, we conclude that LOCE should be included in the assessment and treatment of people with eating disorders.

The findings of this study have relevance for the understanding of the neuro-psycho-modulatory effects of binge eating. It has been argued that the severe levels of high weight found in people with BED and other EDs are due to their distinctive vulnerabilities of impulsiveness, reward dependence, and emotional reactivity and, thus, greater likelihood of LOCE [39]. The importance of improved LOCE and its mid-treatment moderation of HAPIFED outcomes aligns with this. This is alongside an increased risk of physical and mental health comorbidities. Thus, treatments need to address the increased severity and complexity of illness for people with high weight [39]. Indeed, HAPIFED aims to do this with multidisciplinary care, additional modules on appetite regulation, behavioural activation, and greater intensity of dietetic treatment.

The present research has both strengths and limitations. The strengths include the use of a randomised trial to determine the predictors and moderators of treatment outcomes for BED or BN in those with high BMI. Thus, minimal adjustment was required for confounding bias to achieve a baseline balance between the two groups. The instruments, i.e., the EDE, EDE-Q, LOCES, and MHRQoL, were internally consistent (Cronbach’s α ≥ 0.7). As Cronbach’s α was very high (0.92) for LOCES, we verified that there was no repetition of any item for LOCES, thus reducing redundancy for LOCES.

Limitations of this study included the inability to control for sex in the regression models, as males were too infrequent in the sample. There were only four men out of 98 participants, thus limiting generalisability and precluding subgroup analysis. The different treatments may have contributed to the difficulty in predicting treatment outcomes. As the severity of ED symptoms differed between participants, we included the severity of ED symptoms as one of the predictors of treatment outcomes. Thus, a less heterogeneous (with less variability in symptom severity) and larger sample might be more informative in future studies. This study did not find MHRQoL to be significantly associated with higher odds of frequency of binge-eating episodes. This may be a type 2 error for MHRQoL as the association was close to statistical significance (*p*-value = 0.06). The same was observed for the frequency of binge-eating episodes predicting MHRQoL. A subgroup analysis for the largest eating disorder group, i.e., those with BED, was not conducted as the patients were no longer randomly distributed between the treatment and control groups; hence, there was potential for large baseline imbalances between the two groups if a subgroup analysis was performed. As the sample size was only 66 for those with BED, this meant that few potential confounders could be adjusted for in the analysis, which may have resulted in residual confounding bias. Further, a longer follow-up would be advantageous in assessing the long-term stability of treatment effects. LOCE is also an important feature of dysregulation of appetite. Future research could extend the present findings and include an assessment of appetite self-regulation and self-control strategies, e.g., with the use of the TEMPESR self-regulation questionnaire for eating [40], which has been validated in young people at the population level. Finally, our MTLOCE occurred at 3 months from the start of treatment, and we did not have a measure that would inform the effects of early change in therapy, which was a consistent predictor of outcomes.

## 5. Conclusions

MTLOCE predicted and moderated some but not all treatment outcomes. In particular, a greater reduction in MTLOCE increased MHRQoL and decreased the frequency of binge-eating episodes and eating disorder symptom severity. These associations were found independent of the baseline frequency of binge-eating episodes. Moderation of the effect of HAPIFED treatment by MTLOCE, but not baseline weight, decreased both frequency of binge-eating episodes and eating disorder symptom severity. A larger longitudinal study with a greater representation of men is recommended for greater generalisability and replicability of findings.

## Figures and Tables

**Table 1:** The distribution of clinical features and putative predictor variables of the outcomes for participants at baseline.

Predictors	Mean (SE) Total Sample (*n* = 98)
Illness duration/years	14.98 (1.15)
Binge-eating episode frequency	39.40 (3.00)
Eating disorder symptom severity score	2.56 (0.08)
MHRQoL	35.59 (1.10)
Baseline LOCE	3.21 (0.06)
MTLOCE	2.32 (0.09)
Weight (kg)	88.27 (1.27)
Body mass index (kg/m^2^)	33.68 (0.33)
**Predictors**	**Percentage** **(SE)**
Purging behaviour	18.37 (0.04)

Notes: MHRQoL = mental health-related quality of life; MTLOCE = mid-therapy reduction in loss of control over eating (LOCE).

**Table 2:** Results of behavioural correlation of HAPIFED treatment outcomes to 12-month follow-up.

	Clinical Outcomes (Dependent Variables)											
Independent Variables	Binge-Eating Episode Frequency			Eating Disorder Symptom Severity			Body Mass Index			MHRQoL		
	IRR (SE)	95% CI		COEFF (SE)	95% CI		COEFF (SE)	95% CI		COEFF (SE)	95% CI	
Baseline binge-eating episode frequency	1.0048 (0.006)	0.9926	1.0171	0.0044 (0.0045)	−0.0046	0.0133	−0.0061 (0.0078)	−0.0215	0.0092	0.0866 (0.048)	−0.0096	0.1828
Baseline eating disorder symptom severity	1.4814 * (0.178)	1.0413	2.1075	0.3872 * (0.1517)	0.0824	0.6921	−0.4572 (0.3276)	−1.1106	0.1961	0.8708 (1.802)	−2.7182	4.4599
Baseline illness duration (years)	0.9944 (0.017)	0.9613	1.0286	−0.0118 (0.0128)	−0.0371	0.0135	0.0023 (0.0221)	−0.0414	0.0459	−0.0306 (0.1515)	−0.3374	0.2762
Baseline purging behaviour	0.8956 (0.4787)	0.3464	2.316	−0.0326 (0.3414)	−0.7081	0.6429	−0.2118 (0.7286)	−1.6844	1.2607	2.3406 (3.9572)	−5.6180	10.2993
Baseline MHRQoL	1.0269 (0.0153)	0.9963	1.059	−0.0157 (0.0138)	−0.0435	0.0121	0.0303 (0.0250)	−0.0201	0.0808	0.3528 * (0.1362)	0.0789	0.6266
Baseline body mass index	1.008 (0.0144)	0.9794	1.037	0.0062 (0.0109)	−0.0155	0.0279	0.9092 ^ (0.0812)	0.7456	1.0728	−0.0817 (0.4571)	−1.0043	0.8409
MTLOCE	0.5637 * (0.235)	0.3539	0.8977	−0.6505 ^ (0.2115)	−1.0792	−0.2217	−0.3120 (0.3604)	−1.0386	0.4145	5.6232 ^ (1.9584)	1.6877	9.5587

Notes: * *p* ≤ 0.05; ^ *p* ≤ 0.01. Results suppressed for the controls, type of eating disorder diagnosis, HAPIFED therapy group, and age. IRR = incidence rate ratio, COEFF = coefficient; MHRQoL = mental health-related quality of life; MTLOCE = mid-therapy reduction in loss of control over eating. All models are adjusted for body weight except for the BMI model.

**Table 3:** Effect sizes and slopes for the interaction of HAPIFED therapy with MTLOCE by baseline weight and baseline body mass index (BMI).

Outcomes	Interaction	Slope (SE)	Effect Size	Value
Binge-eating episode frequency	HAPIFED × MTLOCE	−0.636 * (0.28)	IRR	0.529
Binge-eating episode frequency	HAPIFED × Baseline Weight	0.009 (0.02)	-	-
Eating disorder symptom severity score	HAPIFED × MTLOCE	−0.268 * (0.13)	Cohen’s d	−0.102
Eating disorder symptom severity score	HAPIFED × Baseline Weight	−0.008 (0.02)	-	-
BMI	HAPIFED × MTLOCE	0.456 (0.75)	-	-
BMI	HAPIFED × Baseline BMI	0.032 (0.15)	-	-
MHRQoL	HAPIFED × MTLOCE	1.944 (4.05)	-	-
MHRQoL	HAPIFED × Baseline Weight	0.208 (0.90)	-	-

Notes: * *p* ≤ 0.05. All predictors are measured at baseline. IRR = incidence rate ratio; BMI = body mass index; MHRQoL = mental health-related quality of life; HAPIFED = healthy approach to weight management and food in eating disorders; MTLOCE = mid-treatment reduction in loss of control over eating.

## Data Availability

The data presented in this study are available on request from the corresponding author as the data are not publicly available due to privacy and ethical restrictions.

## Supplementary Materials

The following supporting information can be downloaded at https://www.mdpi.com/article/10.3390/nu17071288/s1, Table S1: Summary of similarities and differences between HAPIFED and Cognitive Behaviour Therapy—Enhanced therapies as implemented in the present study. Figure S1. Participant recruitment and flow diagram.

## Abbreviations

The following abbreviations are used in this manuscript:

AN	Anorexia Nervosa
BED	Binge-Eating Disorder
BMI	Body Mass Index
BN	Bulimia Nervosa
BWLT	Behavioural Weight Loss Therapy
CBT	Cognitive Behavioural Therapy
CBT-E	Cognitive Behavioural Therapy-Enhanced
CI	Confidence Interval
COEFF	Coefficient
DSM	Diagnostic and Statistical Manual of Mental Disorders
EDE	Eating Disorder Examination
EDE-Q	Eating Disorder Examination Questionnaire
EndFU	6 and 12 months end of follow-up
HAPIFED	Healthy Approach to Weight Management and Food in Eating Disorders
ICD	International Classification of Diseases
IRR	Incidence Rate Ratio
LOCE	Loss of Control Over eating
LOCES	Loss of Control Over Eating Scale
MCMC	Markov Chain Monte Carlo
MHRQoL	Mental Health-Related Quality of Life
MTLOCE	Mid-treatment Reduction in Loss of Control Over Eating
OSFED	Other Specified Feeding or Eating Disorder
PROATA	Eating Disorder Program
RCT	Randomised Controlled Trial
SE	Standard Error
SF-12	The 12-Item Short Form Health Survey
UFED	Unspecified Feeding or Eating Disorder
UNIFESP	Universidade Federal de São Paulo
