# Supplementary material for: Predictors and Moderators of Outcomes in a Trial of Cognitive Behavioural Therapy Integrated with Behavioural Weight Loss for High Weight Individuals with Disorders of Recurrent Binge Eating
Source: Nutrients. 2025 Apr 7;17(7):1288. doi: 10.3390/nu17071288 (PMC11990706; doi:10.3390/nu17071288)
Supplement: Supplementary file 1 [file nutrients-17-01288-s001.zip › nutrients-3552407-supplementary.pdf]

Supplementary Table S1: Summary of similarities and differences between HAPIFED and Cognitive Behaviour Therapy –Enhanced therapies as implemented in the present study.

Included	HAPIFED	CBT-E
**Use of Eating Disorder CBT-E formulation**	Extended to address relationship between binge eating and a high BMI	As in CBT-E manual
**Psychoeducation**	Extended to include the relationship between EDs and high BMI	As in CBT-E manual- focus on ED only
**Nutritional counselling**	Dietician led	Mental health therapist led
**Behavioural monitoring**	Extended to include hunger/satiety ratings	As in CBT-E manual
**Multidisciplinary**	With dietetic and activity therapist	Mental health therapist alone
**Session / duration**	30 sessions/6 months	30 sessions/6 months - extended from the 20 session CBT-E manual to match HAPIFED
**Weight loss management**	Dietetic led nutrition counselling	Not presented
**Behavioural activation**	Promotion of increased activity and healthy exercise versus ED driven exercise	Not presented
**Emotion regulation skills**	Module as in the CBT-E manual	Module as in the CBT-E manual

CBT-E = Cognitive Behaviour Therapy - Enhanced
ED = Eating Disorders
HAPIFED = Healthy APproach to WeIght management and Food in Eating Disorders
BMI = Body mass Index (kg/m^2)

Adapted from Palavras MA, Hay P, Touyz S, Sainsbury A, da Luz F, Swinbourne J, Estella NM, Claudino A. Comparing cognitive behavioural therapy for eating disorders integrated with behavioural weight loss therapy to cognitive behavioural therapy-enhanced alone in overweight or obese people with bulimia nervosa or binge eating disorder: study protocol for a randomised controlled trial. Trials. 2015 Dec;16(1):1-0. Used with author permission under Creative Commons Licence conditions.

Supplementary Figure S1. Participant recruitment and flow diagram.

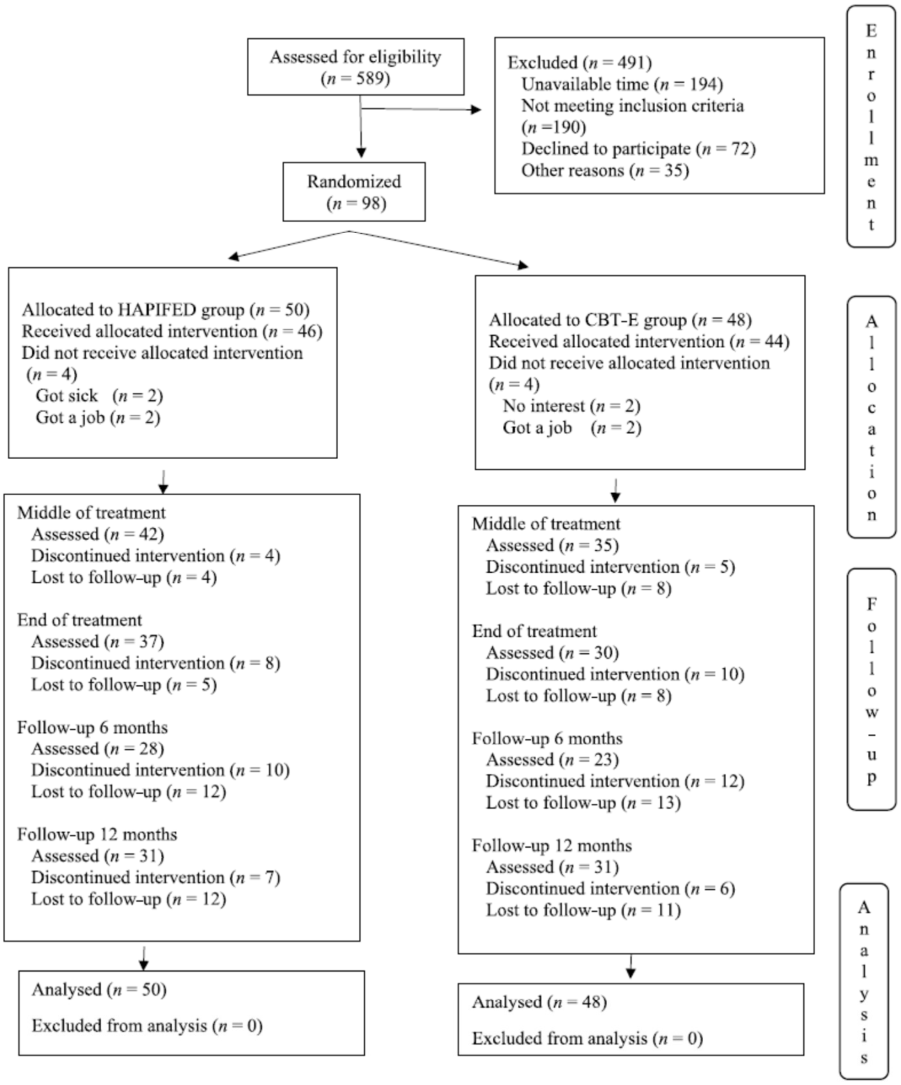

Fig. 1 Participant flow chart. Footnote: Reprinted by permission from Springer Nature Customer Service Centre GmbH, first published in Eat Weight Disorders – Studies on Anorexia, Bulimia and Obesity with the title "Integrated weight loss and cognitive behavioural therapy (CBT) for the treatment of recurrent binge eating and high body mass index: a randomized controlled trial", authors: Palavras MA, Hay P, Mannan H, da Luz FQ, Sainsbury A, Touyz S, Claudino AM. Copyright, 2021. https://doi.org/10.1007/s40519-020-00846-2
